# Supplementary material for: What’s in a Name? An Overview of the Proliferating Nomenclature in the Field of Phage Lysins
Source: Cells. 2023 Aug 7;12(15):2016. doi: 10.3390/cells12152016 (PMC10417350; doi:10.3390/cells12152016)
Supplement: Supplementary file 1 [file cells-12-02016-s001.zip › cells-2519367-supplementary.pdf]

Supplementary Materials to:

What's in a name? An overview on the proliferating nomenclature in the field of phage lysins

Roberto Vázquez, Yves Briers

Supplementary materials contain the transcription and our own English translation of the 1921 communication "Unicité du bacteriophage; Sur la lysine du bacteriophage" by F. D'Herelle and G. Eliava, originally published in *Comptes Rendus de la Société de Biologie et de ses Filiales* (85, 701–702) and retrieved from https://gallica.bnf.fr/edit/und/societe-de-biologie. This is one of the earliest records of the word "lysin" as applied to the phage field, and shows the original distinction between the phage (as a replicative entity) and the lysin (as an enzymatic principle originated in the phage but separated from it).

Comptes Rendus de la Société de Biologie et de ses Filiales, 85, 701–702

SÉANCE DU 22 OCTOBRE

UNICITÉ DU BACTERIOPHAGE ;

SUR LA LYSINE DU BACTÉRIOPHAGE,

Par F. D'HERELLE et G. ELIAVA.

Un serum anti-bacteriophage contient-il une sensibilisatrice pour l'ultramicrobe bacteriophage? Etant donné le serum d'un lapin prepare par une serie d'injections de culture du bactériophage anti-Shiga, la preuve de l'existence, dans ce sérum, d'une sensibilisatrice spécifique pour le bacteriophage anti-dysentérique ne peut guère être faite: en effet, la culture du bacteriophage anti-dysentérique ne peut-être qu'une suspension d'ultramicrobes dans un liquid renfermant la substance dissolute des Bacilles dysentériques aux dépens desquels les ultramicrobes se sont developpés; le séru fourni par l'animal qui a reçu des injections d'une telle culture renferme nécessairement deux sensibilisatrices, l'une spécifique pour le Bacille dysentérique, l'autre pour l'ultramicrobe, et on ne peut séparer les deux actions; on aura toujours une fixation du complement, sans qu'il soit possible de savoir sur lequel des deux antigens presents elle se sera effectuée. La question est pourtant resoluble d'une autre manière bien autrement demonstrative.

L'un de nous a présenté, dans des notes antérieures, diverses expériences démontrant l'unicité du Bactériophage. S'il en est ainsi, la sensibilisatrice contenue dans le sérum anti-Bactériophage-Shiga doit se fixer sur tous les bactériophages, la preuve de la présence d'une sensibilisatrice devient donc posible, car en opérant sur une culture d'un bactériophage autre que l'antidysentérique, rien ne vient plus troubler la réaction. Une culture du Bactériophage anti-pesteux, par exemple, est une suspension d'ultramicrobes dans un liqude contenant en solution la substance des Bacilles pesteux; le seul élément commun dans une culture de Bactériophage anti-dysentérique et dans une culture de Bactériophage anti-pesteux, ne peut être que les ultra-microbes bactériophages; le seul élément anti contenu dans le sérum anti-Bactériophage-Shiga pouvant exercer une action sur une telle culture, ne peut être qu'une sensibilisatrice pour le seul élément commun à toutes les cultures du Bactériophage, les ultra-microbes bactériophages eux-mêmes.

C'est ce que l'expérience demontre; le sérum anti-Bactériophage-Shiga contient une sensibilisatrice spécifique pour le Bactériophage, quelle que soit la souche et quelle que soit l'espèce bactérienne sur laquelle le Bactériophage ait agi. Le Bactériophage est un et le fait vient encore confirmer qu'il s'agit bien d'un être vivant autonome.

La nature Vivante du Bactériophage resort encore des expériences suivantes. L'un de nous a indiqué, à plusieurs reprises, que l'ultra-microbe ne pouvait agir sur le Bactéries que par la sécrétion de diastases dissolvantes, de lysines. Or, nous sommes parvenus à isoler les lysines, libres d'ultramicrobes- Il suffit, pour les obtener, de précipite une culture du bacteriophage par l'alcool: après 48 heures de contact, le précipité contient une substance qui agit sur les

bacteries: il ne peut s'agir que des lysines du bacteriophage. Menionnons de plus que ces lysines sont douées d'un povoir opsonique d'une puissance considerable.

La possibilité d'isoler les lysines, diastases au moyen desquelle le bacteriophage agit, et de séparer ainsi l'action vitale, c'est-à-dire l'action en serie, de l'action lytique elle-même, implique nécessairement la nature vivante du Bacteriophage.

Ce deux series d'expériences viennent une fois de plus démontrer que le Bactériophage est un ultramicrobe parasite des Bactéries, susceptible de s'adapter par accoutumance au parasitisme vis-à-vis des espèces bactériennes les plus variées.

L'espace limité nous empêche de fournir ici les protocoles des expériences justificatives: ils figureront dans un ouvrage d'ensemble sur le Bactériophage, actuellement sous presse.

Proceedings of the Société de Biologie and its Affiliates, 85, 701–702

MEETING OF OCTOBER 22

UNICITY OF BACTERIOPHAGE ;

ON THE LYSIN OF BACTERIOPHAGE,

By F. D'HERELLE and G. ELIAVA.

Does an anti-bacteriophage serum contain an [antibody][1] [against][2] the bacteriophage ultramicrobe? Given the serum of a rabbit prepared by a series of injections of an anti-Shiga[3] bacteriophage culture, the case for the existence of a specific [antibody] [against] the anti-dysenteric bacteriophage in this serum can hardly be made: indeed, the culture of the anti-dysenteric bacteriophage can only be a suspension of ultramicrobes in a liquid containing the dissolute substance of the dysenteric bacilli at expense of which the ultramicrobes have developed; the serum supplied by the animal which has received injections of such a culture necessarily contains two [antibodies], one specific for the dysenteric bacillus, the other for the ultramicrobe, and the two [activities][4]; there would always be a fixation of the complement, without it being possible to know on which of the two antigens it would have taken place. The question can, however, be answered in another and much more demonstrative way.

One of us has presented, in earlier notes, various experiments demonstrating the unicity of the bacteriophage[5]. If this is so, the [antibody] contained in the anti-bacteriophage-shiga serum must bind to all the bacteriophages, the proof of presence of an [antibody] thus becomes possible, because by operating on a culture of a bacteriophage other than the anti-dysenteric, nothing more comes to disturb the reaction. A culture of anti-plague[6] bacteriophage, for example, is a suspension of ultramicrobes in a liquid containing the substance of plague bacilli in solution; the common element in a culture of anti-dysenteric bacteriophage and a culture of anti-plague bacteriophage can only be the bacteriophage ultra-microbes; the only anti[body] element contained in the anti-bacteriophage-Shiga serum that can exert an action on such a culture[7] can only be an [antibody] [against] the only element common to all bacteriophage cultures, the bacteriophage ultra-microbes themselves.

---

[1]"Sensibilisatrice" has been translated as [antibody] throughout the full text. Square brackets have been however added to denote a modification from the original.
[2]We have chosen to translate "sensibilisatrice pour" as "antibody against" instead of "sensitizer for" or "antibody for", to better conform to contemporary language.
[3] Presumably "anti-*Shigella dysenteriae*". Likewise, all subsequent mentions to "Shiga" or "dysentérique" can be understood as the bacterial pathogen *S. dysenteriae*.
[4] "Actions", as translated literally from the original.
[5] Although what the authors exactly mean remains relatively obscure, we believe they intend to prove the independent existence of the bacteriophage as oneself, as a member of the particular set of beings which share, precisely, the feature of being bacteriophages, and, subsequently, not essentially belonging to another class of beings.
[6] Anti-*Yersinia pestis*.
[7] The culture of phages anti-plague.

This is what experience shows; the anti-bacteriophage-Shiga serum contains a specific sensitizer for bacteriophage whatever the strain and whatever the bacterial species on which the bacteriophage has acted. The bacteriophage is one and [this] fact further confirms that it is indeed an autonomous living being.

The living nature of the bacteriophage still resides in the following experiments. One of us has indicated, on several occasions, that the ultramicrobe could only act on the bacteria by the secretion of dissolving [enzymes][8], of lysins. Now, we have succeeded in isolating the lysins, free from ultramicrobes. To obtain them, it is enough to precipitate a culture of bacteriophage with alcohol: after 48 hours of contact the precipitate contains a substance which acts on the bacteria: it can only be the lysins of the bacteriophage. Let us also mention that these lysins are endowed with a considerable opsonic power.

The possibility of isolating the lysins, the [enzymes] by means of which the bacteriophage acts, and of thus separating the vital action, that is to say the serial action, necessarily implies the living nature of the bacteriophage. These two series of experiments demonstrate once more that the bacteriophage is an ultramicrobial parasite of bacteria, capable of adapting itself by habituation to parasitism towards the most varied bacterial species. Space limitations prevent us from providing here the protocols of the supporting experiments: they will appear in a comprehensive book on bacteriophage, currently in press.

[8]We translate "diastase" as "enzyme", understanding that the first enzyme discovered and named (the "diastase") gave its name metonymically to the whole category of entities behaving similarly (all the "enzymes").
